# Lipid Metabolism, Body Composition, and Diet in Acne Vulgaris: A Narrative Review

**DOI:** 10.3390/metabo16070466

**Published:** 2026-07-03

**Authors:** Olivia Jakubowicz-Zalewska, Angelika Biełach-Bazyluk, Hanna Myśliwiec, Mateusz Matwiejuk, Iwona Flisiak

**Affiliations:** Department of Dermatology and Venereology, Medical University of Białystok, 15-540 Białystok, Poland; angelika.bielach-bazyluk@umb.edu.pl (A.B.-B.); hanna.mysliwiec@umb.edu.pl (H.M.); iwona.flisiak@umb.edu.pl (I.F.)

**Keywords:** acne, metabolism, lipid profile, obesity, diet

## Abstract

Acne vulgaris is a common chronic inflammatory disorder of the pilosebaceous unit that is increasingly recognized as a condition linked to systemic metabolic disturbances. Growing evidence suggests that alterations in lipid metabolism, both in sebum composition and circulating lipid profiles, may play a key role in acne pathogenesis. This narrative review aims to summarize current knowledge on the relationships between lipid metabolism, body composition, diet, and acne. Acne vulgaris should be considered not only a dermatological condition but also a disorder with metabolic components. A deeper understanding of lipid-related mechanisms may support the development of more personalized and metabolically targeted therapeutic strategies.

## 1. Introduction

Acne vulgaris is a chronic inflammatory skin disease originating within the pilosebaceous unit. It is one of the most common dermatological disorders worldwide and is estimated to affect nearly one-tenth of the global population [1]. Moreover, its prevalence has increased in recent years [2]. Acne vulgaris is often associated with other systemic diseases or syndromes, including metabolic and endocrine disorders [3]. The clinical presentation of acne is heterogeneous and includes both non-inflammatory lesions, such as open and closed comedones, and inflammatory lesions, including papules, pustules, and nodules [4]. Although acne most commonly develops during adolescence and is more prevalent in young men, it is also increasingly recognized in adulthood, particularly among women. The pathogenesis of acne is multifactorial and involves abnormal follicular keratinisation, excessive sebum production, inflammation, and colonization of hair follicles by *Cutibacterium acnes* (*C. acnes*) [4]. Genetic predisposition and ethnicity contribute to disease susceptibility; however, the increasing prevalence of acne has also been linked to Westernized lifestyle patterns. In particular, diets rich in saturated fats and refined carbohydrates, combined with a low intake of polyunsaturated fatty acids (PUFAs), have been implicated in acne development [5]. Beyond its physical manifestations, acne may substantially impair quality of life. Lesions typically occur in visible areas, including the face, chest, and back, and may result in permanent scarring. Consequently, acne is frequently associated with emotional distress, anxiety, and reduced psychosocial well-being [3].

Although associations between dietary habits and acne have been reported often, the mechanisms linking dietary patterns, circulating lipid fractions, fatty acid composition, and disease expression are less recognized. A better understanding of lipid metabolic pathways may help clarify their role in acne pathogenesis and disease severity.

This review summarizes current evidence regarding the relationships between diet, lipid metabolism, body composition, and acne vulgaris. It examines the potential role of metabolic dysregulation in acne pathogenesis and disease severity and discusses the impact of isotretinoin therapy on systemic and cutaneous lipid metabolism.

## 2. Materials and Methods

A medical literature search of PubMed (1978–present) was conducted in February 2026 using appropriate terms without date limitations. The primary objective of the search was to identify studies investigating lipid metabolism, body composition, dietary factors, and isotretinoin-associated metabolic alterations in acne vulgaris. Medical Subject Headings terms included acne vulgaris, diet, obesity, body composition, and isotretinoin. To ensure comprehensive coverage of the available evidence, additional keywords were applied, including omega-3, omega-6, adipose tissue, leptin, adiponectin, lipid, cholesterol, and lipidomics.

A narrative review methodology was selected to provide an integrative overview of the complex interactions among dietary factors, lipid metabolism, body composition, adipokine signalling, and acne vulgaris. Given the substantial heterogeneity in study design, populations, outcomes, and investigated metabolic parameters, a systematic review or meta-analysis was not considered appropriate. Studies were synthesized narratively. The findings were organized into major themes, including sebum composition, systemic lipid metabolism, fatty acid signalling, body composition, adipokines, dietary influences, isotretinoin-associated metabolic effects, and metabolomic and lipidomic findings.

Studies were considered eligible if they evaluated associations between acne vulgaris and lipid metabolism, body composition, adipokines, dietary factors, fatty acid metabolism, or isotretinoin-associated metabolic effects. Original studies, clinical trials, observational studies, systematic reviews, and relevant mechanistic studies published in English were considered for inclusion. Previously published narrative reviews were considered as supplementary sources to provide contextual background and facilitate the identification of additional relevant publications. Exclusion criteria were as follows: studies using animal models, editorials, and non-peer-reviewed sources; studies focusing on dermatological conditions other than acne vulgaris, unless acne-specific analyses were reported. Reference lists of relevant publications were manually screened to identify additional studies not captured by the database search.

Afterwards, the titles and abstracts of the identified papers were independently screened by two reviewers (O.J.-Z. and A.B.-B.) in order to identify relevant studies to the objectives of the review. Disagreements between reviewers were resolved by a third reviewer (H.M.). Full-text articles meeting the inclusion criteria were further evaluated for their methodological relevance and contribution to the thematic scope of the review. The final set of studies was approved by all authors and incorporated into the narrative synthesis. In total, we included 51 articles for full-text assessment. These studies constituted the core evidence base of the narrative review. In addition, supplementary publications were identified through manual screening of reference lists and were included when they provided relevant background information, expanded specific topics, or offered mechanistic insights relevant to the scope of the review.

**Figure 1:**
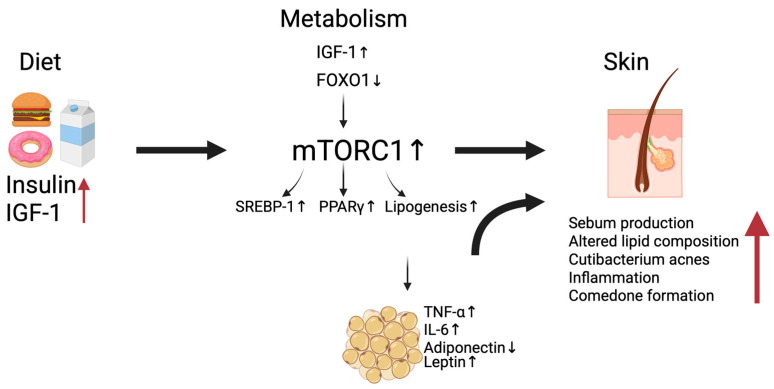
The potential pathogenesis of acne, metabolic disturbances, and diet. Western dietary patterns characterized by high-glycaemic-load foods and dairy intake increase insulin and IGF-1 signalling, resulting in reduced nuclear FOXO1 activity and subsequent activation of mTORC1. mTORC1 stimulates sebaceous lipogenesis through upregulation of SREBP-1 and PPARγ. In parallel, obesity and adipose tissue dysfunction may promote systemic inflammation via adipokine imbalance, increased free fatty acid release, and elevated production of pro-inflammatory cytokines. These interconnected metabolic and inflammatory pathways alter sebum composition, enhance sebaceous gland activity, promote follicular hyperkeratinisation, and may contribute to the development and progression of acne vulgaris. The molecular effects of insulin/IGF-1 signalling on FOXO1, mTORC1, SREBP-1, and sebaceous lipogenesis are supported by experimental studies. In contrast, the links between obesity, adipokine dysregulation, systemic lipid abnormalities, and acne severity are based mainly on associative clinical and epidemiological evidence and should be interpreted as potential contributing mechanisms rather than established causal pathways. IGF-1—Insulin-like Growth Factor-1; FOXO1—Forkhead Box Protein O1; mTORC1—Mechanistic Target of Rapamycin Complex 1; SREBP-1—Sterol Regulatory Element-Binding Protein 1; PPARγ—Peroxisome Proliferator-Activated Receptor Gamma. The work is based on previous studies [40,42,52,54]. Created with BioRender.com.

A schematic illustration summarizing the proposed interactions between diet, metabolic disturbances, and acne vulgaris pathogenesis (Figure 1) was created using BioRender.com.

## 3. Results and Discussion

### 3.1. Alterations in Sebum Composition and Their Role in Acne Vulgaris

The development of acne lesions begins with the activation of inflammatory processes within the pilosebaceous unit [6]. The human sebaceous gland is an exocrine gland composed predominantly of sebocytes and is most densely distributed on the scalp, face, upper chest, and back [7]. Sebaceous glands develop in parallel with the hair follicle within the mid-dermis and empty into the follicular canal, which ultimately opens onto the skin surface [7]. Although the number of sebaceous glands remains relatively constant throughout life, their size increases with age [7,8].

Sebaceous glands consist of undifferentiated, differentiating, and mature sebocytes. Differentiating sebocytes synthesize sebum, which accumulates in cytoplasmic lipid droplets prior to holocrine secretion [9]. Sebaceous gland activity begins at birth and re-emerges during adrenarche, primarily under the stimulatory influence of androgens [6]. In addition to androgen-mediated regulation, sebocyte proliferation and sebum production are modulated by peroxisome proliferator-activated receptor (PPAR) ligands, retinoids, vitamin D, and liver X receptor (LXR) ligands [6,10]. PPARγ plays a central role in sebocyte differentiation and lipogenesis. In addition, activation of PPARγ has been shown to exert anti-inflammatory effects by downregulating the expression of pro-inflammatory cytokines and modulating innate immune responses in sebocytes [6].

Sebaceous glands secrete lipids that are synthesized largely de novo [11]. Sebum is composed predominantly of triglycerides, diglycerides, free fatty acids, wax esters, squalene, and cholesterol [6]. Its composition varies between individuals and is influenced by age [11]. The primary functions of sebum include lubrication of the skin and hair, maintenance of epidermal hydration, antimicrobial activity, and participation in innate immune responses [6].

Increasing evidence suggests that qualitative alterations in sebum lipid composition may be more important in acne pathogenesis than sebum overproduction alone [11]. In particular, alterations in the composition of essential fatty acids have been increasingly implicated in acne pathogenesis. Reduced levels of linoleic acid have been consistently observed on the skin surface and within wax esters of patients with acne [12]. Given that linoleic acid plays a key role in sebaceous lipid synthesis and epidermal barrier maintenance, its deficiency may promote comedone formation and increase susceptibility to local inflammation [13]. Among the lipid alterations implicated in comedogenesis, elevated levels of free fatty acids and squalene peroxidation products have been identified [12]. Furthermore, polar lipids derived from oxidized squalene have been shown to stimulate keratinocyte proliferation, suggesting a pathogenic role of squalene oxidation in lesion development [14]. Squalene is highly susceptible to oxidation by ultraviolet radiation and environmental pollutants, resulting in oxidation products, such as squalene monohydroperoxide. Squalene monohydroperoxide exerts potent pro-inflammatory effects by stimulating the release of cytokines such as interleukin-6 (IL-6) and activating lipoxygenase (LOX)-dependent pathways. LOX enzymes catalyze the oxidation of PUFAs, generating lipid mediators involved in inflammatory processes. Emerging evidence suggests that *C. acnes* may possess the ability to promote squalene oxidation within the pilosebaceous unit, thereby contributing to the formation of pro-inflammatory lipid oxidation products. Collectively, these processes may subsequently promote follicular hyperkeratinisation, comedogenesis, and acne progression [15].

Additionally, altered palmitic acid-to-sapienic acid ratios in triglycerides and wax esters have been reported in the sebum of patients with acne [12]. Lower levels of essential fatty acids have also been observed in acne-associated sebum [11]. Furthermore, recent studies have identified alterations in the relative proportions of saturated and monounsaturated fatty acids [12]. Collectively, these findings may reflect disturbances in fatty acid desaturation and lipid remodelling pathways; however, their precise contribution to acne pathophysiology remains only partially understood. It is currently unclear whether these alterations play a direct role in acne development or represent secondary consequences of sebaceous gland dysfunction.

Disturbances in sebum lipid homeostasis may further contribute to acne pathogenesis by modulating microbial colonization and inflammatory signalling. Alterations in sebum lipid composition shape the local microbial ecosystem of the pilosebaceous unit. A sebum-rich environment supports the growth of lipophilic microorganisms, particularly *C. acnes. C. acnes* utilizes sebum lipids as a primary energy source. Through the activity of bacterial lipases, sebaceous triglycerides are hydrolyzed into free fatty acids (FFAs), which may further amplify local inflammatory responses and modify follicular lipid composition. Consequently, the bidirectional interaction between sebum lipid composition and the cutaneous microbiome represents a pivotal component in the pathogenesis of acne [11,16]. Emerging evidence suggests that acne is associated not with an overall increase in *C. acnes* abundance, but rather with a loss of microbial diversity and a shift toward acne-associated *C. acnes* phylotypes, resulting in cutaneous dysbiosis [17]. Changes in sebum quantity and composition may therefore influence microbial homeostasis and promote inflammatory signalling within the pilosebaceous unit. This bidirectional interaction between host lipid metabolism and the skin microbiome represents an important component of acne pathophysiology and may constitute a potential therapeutic target.

### 3.2. Association Between Serum Lipids and Acne Severity

Growing evidence indicates that acne vulgaris may be associated with disturbances in systemic lipid metabolism. Several studies have reported higher serum concentrations of total cholesterol (TC), triglycerides (TGs), low-density lipoprotein (LDL), and lipoprotein (a) in patients with acne when compared with healthy controls [18,19,20,21,22]. In addition, some investigations have reported correlations between elevated TC, TG, and LDL levels and increasing acne severity; whereas high-density lipoprotein (HDL) concentrations tended to be lower in patients with more severe disease [21,22]. Findings across studies remain inconsistent. While some authors reported significant differences in multiple lipid fractions [19,21,22], others observed elevations only in selected lipid parameters or failed to detect statistically significant differences between patients and controls [18].

Despite these limitations, the available evidence suggests a possible association between acne severity and an unfavourable lipid profile characterized by higher TG, TC, and LDL levels and lower HDL concentrations [5].

Similarly, Akaza et al. reported significantly higher triglyceride concentrations in patients with acne compared with healthy controls [23]. The clinical and biological significance of this finding remains uncertain. While elevated triglyceride levels have been associated with acne severity, current evidence does not establish a causal relationship. It has been hypothesized that triglycerides may penetrate the follicular environment, become incorporated into local metabolic pathways, and potentially contribute to comedone formation [24].

These discrepancies may reflect differences in study design, sample size, sex distribution, age, dietary habits, and ethnicity. Another source of heterogeneity is the use of different acne grading systems across studies, which may limit direct comparisons and contribute to inconsistent associations between lipid parameters and disease severity. Most available studies are cross-sectional and observational in nature, precluding conclusions regarding causality. Consequently, it remains unclear whether lipid abnormalities contribute directly to acne pathogenesis or represent secondary manifestations of underlying metabolic disturbances.

### 3.3. Role of Fatty Acids in Acne-Associated Inflammation

Alterations in the fatty acid composition of sebum have been implicated in the initiation of inflammation and activation of innate immune responses within the pilosebaceous unit. One lipid species of particular interest is squalene, which has been shown to promote inflammatory signalling through activation of LOX pathways and increased production of IL-6 [11]. Oxidized squalene derivatives and FFAs may further contribute to inflammation by stimulating keratinocytes to release pro-inflammatory cytokines, including interleukin-1α (IL-1α), interleukin-8 (IL-8), and tumour necrosis factor-α (TNF-α), resulting in follicular hyperkeratinisation and comedone formation [25]. Additionally, oleate has been shown to increase IL-1α mRNA expression. In contrast, linoleate may exert protective effects against acne development, and reduced levels of linoleate have been consistently observed in patients with acne. Together with other sebaceous lipids, these compounds contribute to the maintenance of the epidermal barrier and may help protect against microbial colonization [11].

Lipoprotein lipase (LPL) and secretory phospholipase A_2_ (sPLA_2_), both expressed within sebaceous glands, contribute to the local generation of FFAs. This may amplify inflammatory responses relevant to acne pathogenesis [26]. PUFAs, particularly omega-3 fatty acids, can serve as alternative enzymatic substrates and promote the production of specialized pro-resolving mediators (SPMs), lipid-derived molecules involved in the active resolution of inflammation [27]. Consequently, dietary modulation of fatty acid intake has been proposed as a potential mechanism influencing both sebaceous lipid composition and inflammatory signalling pathways associated with acne. However, the clinical significance of these mechanisms remains unknown, and evidence from human studies remains limited.

LPL classically hydrolyses triglycerides from triglyceride-rich lipoproteins at the luminal surface of capillary endothelial cells. LPL expression has also been demonstrated in skin appendages, suggesting that sebaceous glands may actively acquire circulating fatty acids, potentially linking systemic lipid metabolism with local cutaneous processes [26]. The activity of LPL is tightly regulated by angiopoietin-like proteins 3 and 4 (ANGPTL3 and ANGPTL4), key modulators of plasma lipid homeostasis through inhibition of LPL activity [26]. Secretory phospholipase A_2_ (sPLA_2_), in turn, catalyzes the hydrolysis of membrane phospholipids, releasing FFAs and lysophospholipids, both of which may participate in inflammatory signalling pathways [26]. Aslan et al. reported increased sPLA_2_ activity in patients with acne, accompanied by elevated arachidonic acid (AA)-to-eicosapentaenoic acid (EPA) and dihomo-gamma-linolenic acid (DGLA)-to-EPA ratios in serum. Increased LPL activity was observed in the same cohort [26]. These findings suggest that altered lipid-processing enzyme activity may contribute to changes in local fatty acid availability and inflammatory signalling. To date, these observations have not been consistently replicated in larger cohorts, and their clinical relevance remains to be established.

### 3.4. Role of Omega-3 and Omega-6 Fatty Acids in Inflammatory Pathways and Acne Pathogenesis

Beyond conventional serum lipid parameters, recent attention has been directed toward qualitative alterations in lipid composition [1,2,19,20,26,28,29]. Emerging evidence suggests that specific lipid species and their relative proportions, particularly the omega-6-to-omega-3 and PUFAs-to-monounsaturated fatty acids (MUFAs) ratios—may be associated with acne susceptibility [30]. These lipid profiles may partially reflect dietary patterns characteristic of the Western diet, including increased consumption of saturated fats, high-glycaemic-load foods and dairy products. Such diets have been linked to the activation of inflammatory and lipogenic signalling pathways relevant to acne pathogenesis [31].

Several studies have reported an association between omega-3 supplementation and clinical improvement of acne lesions [18,29,32,33] and beneficial modulation of gut microbial diversity [34]. Omega-3 fatty acids have also been shown to mitigate selected adverse effects of systemic isotretinoin therapy [2]. However, the available evidence remains limited by small sample sizes, heterogeneous study designs, differences in fatty acid sources and dosages, and inconsistent outcome measures. Consequently, the magnitude and clinical relevance of these effects remain uncertain.

In contrast to omega-3 fatty acids, the role of omega-6 fatty acids appears less clearly defined. While supplementation of gamma-linolenic acid (GLA) has shown potential benefits in improving acne lesions [35], other studies have reported associations between higher DGLA levels and increased disease severity [29]. These apparently conflicting findings may reflect the diverse biological functions of omega-6-derived lipid mediators rather than uniform pro-inflammatory effects. Moreover, increasing evidence suggests that the balance between omega-6 and omega-3 fatty acids, rather than the absolute concentration of individual fatty acids, may be a more important determinant of inflammatory responses.

Both AA and EPA participate in inflammatory and inflammation-resolving lipid mediator pathways, and their relative balance may be more relevant than their absolute concentrations [29]. Nevertheless, patients with acne have been reported to exhibit elevated AA-to-EPA and DGLA-to-EPA ratios, suggesting a shift toward a more pro-inflammatory lipid profile [26]. Although these observations support a potential role for PUFA imbalance in acne pathophysiology, current evidence remains largely observational, and causal relationships have yet to be established.

In addition, genetic variability within the fatty acid desaturase (FADS) genes, FADS1 and FADS2, may further influence PUFA metabolism. Zhang et al. identified several single nucleotide polymorphisms (SNPs) within these genes that were associated with altered desaturase activity and potentially contribute to individual differences in acne susceptibility and disease expression [29]. These observations highlight the potential relevance of lipidomic and genomic approaches for improving our understanding of acne pathophysiology. Nevertheless, their clinical application remains largely investigational, and additional studies are needed before such approaches can be incorporated into personalized acne management strategies [36].

However, the available evidence remains limited and heterogeneous, with most studies relying on observational designs that preclude causal inference. Furthermore, it remains unclear whether these lipid alterations contribute directly to acne development or represent secondary manifestations of broader metabolic disturbances. Interpretation of the available evidence is further complicated by substantial heterogeneity among studies, including differences in dietary assessment methods, sources of omega-3 and omega-6 fatty acids, supplementation regimens, dosages, and treatment duration. Moreover, accumulating evidence suggests that the overall balance between omega-6 and omega-3 fatty acids may be more relevant than the intake of individual fatty acids alone, highlighting the importance of evaluating dietary fatty acid ratios rather than isolated nutrients. Consequently, further large-scale prospective and mechanistic studies are needed to clarify the biological significance of these associations. A summary of the major metabolic factors involved in acne vulgaris, their proposed mechanisms, and the corresponding level and type of evidence is presented in Table 1.

### 3.5. Systemic Metabolic Dysregulation in Acne Vulgaris

#### 3.5.1. Obesity and Acne

Overweight is defined as a body mass index (BMI) of 25–29.9 kg/m^2^, whereas obesity is classified as a BMI ≥30 kg/m^2^. The prevalence of obesity continues to rise worldwide, making it a major global health concern associated with cardiovascular complications, diabetes mellitus, and hypertension. Obesity has also been investigated as a potential factor associated with acne vulgaris. While several studies have reported positive associations between increased BMI and acne prevalence or severity, findings remain inconsistent across populations and age groups. Therefore, the epidemiological relationship between obesity and acne has not yet been fully established [37]. Furthermore, BMI may not accurately reflect metabolically active adipose tissue, which could be more relevant to the inflammatory pathways implicated in acne pathogenesis. In this context, assessment of total body fat beyond BMI, particularly visceral adiposity, may better capture the metabolic and inflammatory processes potentially involved in acne development. Accordingly, obesity should currently be regarded as a potential contributing factor rather than an established independent determinant of acne severity.

Several biological mechanisms have been proposed to explain a potential link between obesity and acne. In particular, obesity is characterized by insulin resistance, adipokine imbalance, inflammation, and altered lipid metabolism. All of these factors may influence pathways implicated in acne pathogenesis. In addition, some studies suggest that overweight and obese individuals may exhibit increased sebum production, although the underlying mechanisms remain unclear [37].

Mechanistic target of rapamycin complex 1 (mTORC1) plays a central role in the development and progression of metabolic disorders, including obesity, by promoting lipogenesis through activation of the sterol regulatory element-binding protein 1 (SREBP-1) [38]. This pathway has also been implicated in acne pathophysiology. In sebocytes, mTORC1 stimulates the expression of PPARγ and sterol regulatory element-binding protein-1c (SREBP-1c), the predominant SREBP-1 isoform involved in sebocyte lipogenesis, thereby enhancing sebaceous lipid synthesis. Furthermore, SREBP-1c upregulates stearoyl-CoA desaturase and Δ6-desaturase, promoting the synthesis of MUFAs in sebaceous lipids [39].

Obesity is also associated with alterations in immune function, particularly within white adipose tissue, which may influence T helper 17 (Th17) cell differentiation and activity. Compared with individuals of normal body weight, obese subjects have been reported to exhibit increased numbers of Th17 cells and enhanced Th17 signalling pathways, mechanisms that have also been implicated in acne pathogenesis [37]. In particular, Th17 cells are a major source of interleukin-17 (IL-17), a pro-inflammatory cytokine known to promote keratinocyte proliferation and hyperkeratinisation and may therefore contribute to comedogenesis [48].

Obesity has also been associated with alterations in gut microbiota composition, including reduced microbial diversity and an increased abundance of pro-inflammatory bacterial taxa [49]. Such changes may impair intestinal homeostasis and contribute to systemic inflammation. Furthermore, adipose tissue serves as an important source of FFAs, which may stimulate inflammatory signalling pathways and promote insulin resistance [50].

Insulin resistance is frequently observed in obesity and is often accompanied by compensatory hyperinsulinemia [51]. Elevated insulin levels may stimulate androgen production and increase the bioavailability of IGF-1. Both pathways have been implicated in acne pathogenesis through their effects on sebaceous gland activity, lipogenesis, and keratinocyte proliferation [52]. Furthermore, overweight and obese individuals may exhibit higher circulating androgen levels, which could contribute to increased sebum production and enhanced sebaceous gland activity [37]. Together, these endocrine and metabolic alterations represent plausible mechanisms linking obesity with acne. However, most evidence supporting these associations is indirect and derives from observational or experimental studies. Consequently, it remains uncertain to what extent obesity independently contributes to acne development and severity. Finally, obesity-related metabolic and inflammatory alterations are closely linked to adipokine dysregulation, which is discussed in the following section.

#### 3.5.2. Adipokines

Adipokines are bioactive mediators secreted by adipose tissue that regulate inflammatory responses, energy homeostasis, and insulin sensitivity, suggesting a potential protective role against metabolic and inflammatory disturbances [50]. Adipose tissue secretes a variety of pro-inflammatory mediators, including leptin, TNF-α, and IL-6. Leptin can further stimulate macrophages, monocytes, and T lymphocytes, thereby enhancing cytokine production and inflammatory responses. Conversely, circulating adiponectin levels are typically reduced in obesity. Among them, adiponectin and leptin have attracted particular interest due to their potential involvement in pathways relevant to acne pathogenesis [53,54]; however, available data remain limited and inconsistent.

Adiponectin is an adipocyte-derived hormone predominantly produced by subcutaneous adipose tissue. It exerts anti-inflammatory, antioxidant, and insulin-sensitizing effects. Adiponectin suppresses the production of pro-inflammatory cytokines while promoting anti-inflammatory cytokine secretion. In addition, it downregulates adhesion molecule expression, inhibits Toll-like receptors signalling, and improves insulin sensitivity [55]. At the molecular level, adiponectin inhibits mTORC1 activity through activation of AMP-activated protein kinase (AMPK). In the general population, circulating adiponectin concentrations are inversely associated with BMI [40]^.^ Furthermore, high-glycaemic-load diets have been linked to lower adiponectin levels [56]. Reduced adiponectin concentrations have also been associated with insulin resistance, type 2 diabetes mellitus, and atherosclerosis [55,57]. Several studies have reported lower adiponectin levels in patients with acne compared with healthy controls. However, these findings should be interpreted with caution, as the available studies are characterized by relatively small sample sizes, predominantly involve young adults, and differ substantially with regard to study design, acne severity assessment, and participant characteristics. Consequently, it remains unclear whether reduced adiponectin levels contribute directly to acne pathogenesis or represent a marker of broader metabolic disturbances. Further large-scale prospective studies are required to clarify the clinical significance of these associations.

In contrast to adiponectin, leptin is an adipocyte-derived hormone whose circulating levels increase with adipose tissue expansion. Although the precise role of leptin in the skin and the pathogenesis of dermatological diseases remains incompletely understood, leptin is widely recognized as a pro-inflammatory mediator. However, data regarding serum leptin concentrations in patients with acne are limited and inconsistent.

For example, Ozguz et al. [53] reported significantly altered leptin and adiponectin levels in patients with acne compared with healthy controls, whereas Kaymak et al. [54] did not observe such an association. These discrepancies may be related to differences in study design, sample size, participant characteristics, acne severity, and the presence of metabolic confounders, including BMI and insulin resistance.

Given the opposing biological effects of leptin and adiponectin, the leptin-to-adiponectin ratio has been proposed as a potential marker of obesity and metabolic dysfunction and has been investigated in conditions such as atherosclerosis and type 2 diabetes mellitus [53]. Whether this ratio has clinical relevance in acne remains uncertain.

Overall, the available evidence suggests a possible association between adipokine dysregulation and acne. However, current findings remain inconsistent and are largely derived from relatively small observational studies. Consequently, it remains unclear whether alterations in adipokine levels contribute directly to acne pathogenesis or reflect broader metabolic disturbances. Further large-scale prospective studies are needed to clarify the role of adipokines in acne and their potential utility as metabolic biomarkers.

### 3.6. The Diet–Metabolism–Skin Axis in Acne Development

Diet has emerged as a potentially modifiable factor in acne pathogenesis through its effects on endocrine signalling, insulin sensitivity and inflammatory pathways. Although the available evidence remains heterogeneous and is derived largely from observational studies, several investigations have reported associations between dietary patterns and acne occurrence or severity. In particular, Western dietary patterns, characterized by a high intake of processed foods, refined carbohydrates, and saturated fatty acids, have been linked to increased acne prevalence and severity [58].

High-glycaemic-index foods induce rapid postprandial increases in blood glucose and insulin concentrations, which may subsequently elevate circulating levels of IGF-1. Increased IGF-1 signalling has been implicated in acne pathophysiology through its effects on androgen synthesis, sebaceous gland activity, and lipogenesis [39]. Observational studies have further suggested that acne symptoms may improve during periods of caloric restriction, although the underlying mechanisms have not been fully elucidated [5].

In addition, IGF-1 promotes androgen production while reducing the synthesis of insulin-like growth factor–binding proteins 1 and 3 (IGFBP-1 and IGFBP-3) and sex hormone–binding globulin (SHBG). Reduced concentrations of these binding proteins increase the bioavailability of both IGF-1 and androgens, thereby potentially enhancing sebaceous lipid production and acne-associated signalling pathways [59]. Elevated insulin and IGF-1 signalling have also been linked to follicular hyperkeratinisation and comedogenesis, processes considered central to acne development [48].

Insulin and IGF-1 have been shown to reduce nuclear levels of Forkhead Box Protein O1 (FOXO1) by promoting its translocation from the nucleus to the cytoplasm. In its nuclear form, FOXO1 functions as a transcriptional regulator that suppresses protein synthesis, cell growth, and lipid metabolism. FOXO1 also upregulates the expression of sestrin 3, a negative regulator of mTORC1 signalling. Consequently, FOXO1 nuclear exclusion may result in increased mTORC1 activity and enhanced anabolic signalling, which has been implicated in sebaceous gland proliferation and lipogenesis [39]. Chronic mTORC1 overactivation has also been associated with obesity, metabolic syndrome, and type 2 diabetes mellitus [60].

Dairy products, particularly milk, have likewise been implicated in acne pathophysiology through their potential effects on insulin and IGF-1 signalling, thereby activating pathways similar to those induced by high-glycaemic-load foods [41]. Several observational studies and meta-analyses have reported positive associations between dairy consumption and acne occurrence or severity. However, the available evidence remains heterogeneous, and randomized controlled trials evaluating the effects of dairy restriction on acne outcomes are currently lacking [42,43].

Taken together, these observations support the hypothesis of a diet–metabolism–skin axis linking nutritional factors with endocrine signalling, lipid metabolism, and inflammatory pathways relevant to acne. Nevertheless, much of the available evidence is observational, and the relative contribution of individual dietary factors remains difficult to determine because of substantial heterogeneity in study design, dietary assessment methods, and potential confounding variables. Therefore, while dietary factors may influence pathways implicated in acne pathogenesis, further well-designed prospective and interventional studies are needed to establish causality and to define the clinical relevance of dietary modification in acne management. An overview of the proposed mechanisms linking diet, metabolism, and acne vulgaris is shown in Figure 1.

### 3.7. Isotretinoin and Lipid Metabolism

Oral isotretinoin remains one of the most effective therapeutic options for moderate to severe acne. Although its precise mechanism of action has not been fully elucidated, isotretinoin induces apoptosis of sebaceous gland cells, leading to a marked reduction in sebum production. In addition, it decreases follicular hyperkeratinisation and comedone formation. Through these effects, isotretinoin indirectly suppresses the growth of *C. acnes* and attenuates acne-associated inflammation [44,45]. Common adverse effects include xerosis, cheilitis, facial erythema, and dry lips, with mucocutaneous symptoms representing the most frequently reported treatment-related complications [46].

Beyond its dermatological effects, considerable attention has been focused on the effects of isotretinoin on lipid metabolism and cardiometabolic parameters. Several studies have reported treatment-associated alterations in serum lipid profiles, although the magnitude of these changes varies across populations. Saklamaz et al. demonstrated significant increases in total cholesterol and LDL concentrations during therapy, accompanied by modest elevations in HOMA-IR (Homeostasis Model Assessment of Insulin Resistance) and carotid intima-media thickness, a recognized marker of subclinical atherosclerosis [47]. Similarly, other investigators reported increases in total cholesterol, triglycerides, and LDL levels together with reductions in HDL concentrations [61]. In another study, triglyceride levels increased progressively during treatment, whereas elevations in LDL and total cholesterol were most pronounced during the first two months of therapy [62]. Despite these observations, the clinical significance of isotretinoin-induced dyslipidemia remains uncertain. Although elevations in total cholesterol and triglyceride levels have been consistently reported, most studies describe mild and transient abnormalities that are not associated with significant cardiovascular morbidity in otherwise healthy individuals [63]. Hypertriglyceridemia represents the most clinically relevant metabolic adverse effect of isotretinoin therapy; however, severe elevations requiring treatment modification appear to be relatively uncommon. Park et al. observed a progressive increase in serum triglyceride concentrations during isotretinoin therapy. The median baseline triglyceride level was approximately 70 mg/dL, rising to a peak of approximately 112 mg/dL at the third month of treatment. Notably, although triglyceride concentrations decreased slightly after reaching their peak at month 3, they remained consistently higher than baseline values throughout months 5–6 of therapy, with a statistically significant elevation compared with pretreatment levels [62]. Although severe hypertriglyceridemia is uncommon, triglyceride concentrations exceeding 500 mg/dL generally warrant closer monitoring and consideration of dose reduction or treatment interruption because of the risk of pancreatitis. Furthermore, available evidence suggests that lipid abnormalities generally resolve following treatment discontinuation [44]. Current clinical practice supports assessment of baseline lipid parameters before treatment initiation, followed by periodic monitoring during therapy, particularly in patients with pre-existing dyslipidemia, obesity, insulin resistance, metabolic syndrome or a family history of lipid disorders. Although intensive laboratory monitoring may not be necessary in all low-risk individuals, regular lipid assessment remains advisable in selected patient populations.

Experimental studies indicate that isotretinoin suppresses sebocyte lipogenesis by downregulating SREBP-1-dependent transcriptional pathways and reducing the expression of enzymes involved in fatty acid synthesis. Consequently, isotretinoin may partially reverse the lipogenic phenotype characteristic of acne-prone sebaceous glands [64]. The metabolic effects of isotretinoin therefore provide an interesting model illustrating the distinction between local sebaceous lipid metabolism and systemic lipid homeostasis. While isotretinoin markedly reduces sebaceous lipogenesis and normalizes sebum composition, it may simultaneously induce transient elevations in circulating lipid fractions, suggesting that local and systemic lipid metabolism are interconnected yet regulated through partially independent mechanisms. Emerging evidence further suggests that isotretinoin may influence metabolic pathways beyond lipid metabolism. Several studies have reported transient increases in HOMA-IR and alterations in adipokine concentrations during treatment; however, findings remain inconsistent, and their clinical relevance has yet to be established [47].

Genetic factors may also modulate susceptibility to isotretinoin-associated metabolic disturbances. In particular, the rs7799039 polymorphism within the leptin gene has been associated with greater increases in serum cholesterol concentrations during therapy, highlighting the potential contribution of individual genetic variability to treatment response [65]. Nevertheless, the available evidence remains limited and requires validation in larger prospective studies.

Taken together, isotretinoin exerts profound effects on both sebaceous gland biology and systemic metabolic pathways. While it effectively suppresses sebaceous lipogenesis and partially normalizes acne-associated alterations in sebum composition, it may also induce transient changes in circulating lipid parameters. These observations highlight the complex relationship between local and systemic lipid metabolism in acne vulgaris. However, the long-term clinical significance of these metabolic alterations remains incompletely understood and warrants further investigation.

## 4. Metabolomic and Lipidomic Approaches in Acne Vulgaris

Metabolomics is an emerging field that integrates information from genomics, transcriptomics, proteomics, and metabolite profiling to provide a comprehensive assessment of biological processes. It aims to characterize small molecules and metabolic responses associated with genetic, environmental, and external influences. Among the metabolites analyzed, lipids have attracted particular attention due to their central role in acne pathophysiology. However, metabolomic analyses remain limited by challenges such as incomplete metabolite coverage, variable detection sensitivity, and limited quantitative accuracy [66].

Among metabolomic investigations, lipid-related alterations have been consistently reported, highlighting the close interrelationship between metabolomics and lipidomics in acne research. Several studies applied metabolomic approaches to investigate acne vulgaris. Using LC–MS/MS (Liquid Chromatography-Tandem Mass Spectrometry) analysis of serum samples, Yu et al. identified 63 significantly altered metabolites in patients with acne. Among these, several lipid-related metabolites, including sphinganine, sphingosine, O-phosphoethanolamine, and sphingomyelin (SM d18:1/18:0), were significantly upregulated [67]. Similarly, Li et al. analyzed serum metabolites in acne patients with and without insulin resistance and identified alterations in multiple lipid species, including 1,2-dioleoyl-sn-glycero-3-phosphatidylcholine and SM (d18:1/18:0). Furthermore, Kyoto Encyclopedia of Genes and Genomes (KEGG) pathway analysis identified the sphingolipid signalling pathway as one of the most significantly enriched metabolic pathways [67]. Notably, these findings highlight that a substantial proportion of metabolomic alterations in acne are lipid-derived, partially overlapping with lipidomic observations.

Lipidomics, a specialized branch of metabolomics, focuses specifically on the structure, function, and biological roles of lipids. Recent lipidomic studies have demonstrated alterations in both cutaneous and circulating lipid metabolites in patients with acne, suggesting that lipid dysregulation may contribute to disease pathogenesis [66]. Specifically, patients with acne have been shown to exhibit increased sebum production, which has been accompanied by elevated levels of several lipid classes, including higher concentrations of cholesterol, unsaturated FFAs, and TGs. Furthermore, shorter ceramide chain lengths have been identified in the sebum of acne patients, potentially disrupting lipid organization within the epidermal barrier [68,69]. Lipidomics analyses have also demonstrated associations between acne severity and specific sebaceous lipid signatures. Higher concentrations of diglycerides (DGs), TGs, and FFAs have been reported in patients with moderate acne compared with individuals experiencing milder disease [70]. These findings are consistent with and extend metabolomic data by providing a more detailed characterization of lipid subclasses and their structural heterogeneity.

Nevertheless, the available evidence remains limited, and findings are not always consistent across populations. In addition, most studies have involved relatively small cohorts and have used different analytical platforms, which may contribute to variability in reported results. Although emerging metabolomic and lipidomic approaches provide valuable insights into the metabolic heterogeneity of acne vulgaris, current evidence remains insufficient to support their routine clinical application. Further large-scale studies are needed to validate potential biomarkers and determine whether these technologies may contribute to personalized treatment strategies in the future.

## 5. Conclusions and Future Directions

Acne vulgaris is increasingly recognized as a condition associated with metabolic and nutritional factors that may influence its pathogenesis and clinical course. Current evidence suggests that dysregulation of lipid metabolism, including alterations in sebum composition, circulating lipid profiles, and fatty acid balance, may contribute to pathways involved in acne development, such as sebaceous gland activation, follicular hyperkeratinisation, and inflammation. In addition, emerging data support the involvement of systemic metabolic pathways, including adipokine signalling, insulin/IGF-1–mTORC1 axis activation, and diet-related modulation of inflammatory and lipid metabolic responses. However, many of the reported associations are based on observational studies, and causal relationships remain incompletely established. The metabolic effects of isotretinoin further highlight the complex interplay between local sebaceous lipid regulation and systemic lipid homeostasis, although the long-term clinical significance of these alterations remains uncertain.

Future well-designed prospective studies integrating clinical, metabolic, nutritional, and lipidomic data are needed to clarify these interactions and to determine whether targeted metabolic interventions may improve acne management and allow stratification of patients into metabolically defined acne endotypes.

## Figures and Tables

**Table 1:** Key metabolic mechanisms involved in acne vulgaris. IGF-1—Insulin-like Growth Factor-1; mTORC1—Mechanistic Target Of Rapamycin Complex 1; TNF-α—Tumour Necrosis Factor Alpha; IL-6—Interleukin-6; TGs—Triglycerides; LDL—Low-Density Lipoprotein; HDL—High-Density Lipoprotein; FFAs—Free Fatty Acids. This table summarizes the major metabolic and inflammatory pathways implicated in acne vulgaris. Dietary factors, obesity-associated adipokine imbalance, dyslipidemia, and alterations in fatty acid composition may contribute to acne development through activation of insulin/IGF-1 signalling, mTORC1, inflammatory cytokine production, and disturbances in sebaceous gland lipid metabolism. These mechanisms collectively promote sebocyte proliferation, lipogenesis, follicular hyperkeratinisation, and inflammatory responses within the pilosebaceous unit. The table also highlights the dual role of isotretinoin therapy, which effectively reduces sebaceous gland activity while potentially inducing alterations in systemic lipid metabolism. Based on [1,2,5,11,12,13,14,15,18,19,20,21,22,23,24,25,26,27,36,37,38,39,40,41,42,43,44,45,46,47]. The qualitative levels of evidence presented in Table 1 represent the authors’ narrative assessment of the available literature, based on the consistency of findings across mechanistic studies, observational and epidemiological investigations, clinical studies, randomized controlled trials, systematic reviews, and meta-analyses. They should not be interpreted as a formal evidence-grading system.

Metabolic Factor	Key Mediators/ Pathways	Effect on Sebaceous Unit	Potential Consequence of Acne	Level of Evidence	Type of Evidence
High-glycaemic-load diet	Insulin, IGF-1, mTORC1	Increased sebocyte proliferation and lipogenesis	Increased sebum production and acne severity	High	Mechanistic and observational studies, randomized controlled trials, and meta-analyses
Dairy consumption	IGF-1, insulin, mTORC1 activation	Increased sebocyte proliferation and lipogenesis	Exacerbation of inflammatory lesions	Moderate	Observational studies and meta-analyses; no interventional elimination trials available
Obesity	TNF-α, IL-6, leptin	Systemic inflammation	Enhanced inflammatory signalling	Low-Moderate	Epidemiological and mechanistic studies
Dyslipidemia	TG, LDL, altered HDL	Altered follicular lipid composition	Increased sebaceous gland activity	Moderate	Observational clinical studies with supportive mechanistic evidence
Increased omega-6-to-omega-3 ratio	Arachidonic acid metabolites	Pro-inflammatory eicosanoid synthesis	Amplified inflammation	Moderate	Mechanistic studies, lipidomic analyses, and small clinical supplementation trials
Altered sebum composition	Squalene oxidation, FFAs	Follicular hyperkeratinisation	Comedogenesis	High	Experimental, mechanistic, lipidomic, and clinical evidence studies
Isotretinoin therapy	Sebocyte apoptosis, altered lipid metabolism	Reduced sebum synthesis	Clinical improvement with potential metabolic side effects	High	Mechanistic and prospective clinical studies, systematic reviews

## Data Availability

No new data were created or analyzed in this study.

## Abbreviations

Alphabetical list of abbreviations used in the manuscript.

Abbreviation	Full Term
AA	Arachidonic Acid
AMPK	AMP-Activated Protein Kinase
ANGPTL3	Angiopoietin-Like Protein 3
ANGPTL4	Angiopoietin-Like Protein 4
BMI	Body Mass Index
*C. acnes*	*Cutibacterium acnes*
DG	Diglyceride
DGLA	Dihomo-Gamma-Linolenic Acid
EPA	Eicosapentaenoic Acid
FADS1	Fatty Acid Desaturase 1
FADS2	Fatty Acid Desaturase 2
FFAs	Free Fatty Acids
FOXO1	Forkhead Box Protein O1
GLA	Gamma-Linolenic Acid
HDL	High-Density Lipoprotein
HOMA-IR	Homeostasis Model Assessment of Insulin Resistance
IGF-1	Insulin-Like Growth Factor 1
IGFBP-1	Insulin-Like Growth Factor-Binding Protein 1
IGFBP-3	Insulin-Like Growth Factor-Binding Protein 3
IL-1α	Interleukin-1 Alpha
IL-6	Interleukin-6
IL-8	Interleukin-8
IL-17	Interleukin-17
KEGG	Kyoto Encyclopedia of Genes and Genomes
LDL	Low-Density Lipoprotein
LC-MS/MS	Liquid Chromatography-Tandem Mass Spectrometry
LOX	Lipoxygenase
LPL	Lipoprotein Lipase
LXR	Liver X Receptor
mTORC1	Mechanistic Target of Rapamycin Complex 1
MUFAs	Monounsaturated Fatty Acids
PPARγ	Peroxisome Proliferator-Activated Receptor Gamma
PUFAs	Polyunsaturated Fatty Acids
SHBG	Sex Hormone-Binding Globulin
SM	Sphingomyelin
SNP	Single Nucleotide Polymorphism
SPMs	Specialized Pro-Resolving Mediators
sPLA2	Secretory Phospholipase A2
SREBP-1	Sterol Regulatory Element-Binding Protein 1
SREBP-1c	Sterol Regulatory Element-Binding Protein 1c
TC	Total Cholesterol
TG	Triglyceride
Th17	T Helper 17 Cell
TNF-α	Tumour Necrosis Factor Alpha

## References

[1] Geng R., Sibbald R.G. (2024). Acne Vulgaris: Clinical Aspects and Treatments. Adv. Skin Wound Care.

[2] Zainab Z., Malik N.A., Obaid S., Malik S., Aftab K., Mumtaz M., Pervez A., Syed Z. (2021). Effectiveness of Oral Omega 3 In Reducing Mucocutaneous Side Effects of Oral Isotretinoin in Patients with Acne Vulgaris. J. Ayub Med. Coll. Abbottabad.

[3] Zouboulis C.C. (2014). Acne as a Chronic Systemic Disease. Clin. Dermatol..

[4] Sutaria A.H., Masood S., Saleh H.M., Schlessinger J. (2025). Acne Vulgaris. StatPearls.

[5] Smith R.N., Mann N.J., Braue A., Mäkeläinen H., Varigos G.A. (2007). A Low-Glycemic-Load Diet Improves Symptoms in Acne Vulgaris Patients: A Randomized Controlled Trial. Am. J. Clin. Nutr..

[6] Del Rosso J.Q., Kircik L. (2024). The Primary Role of Sebum in the Pathophysiology of Acne Vulgaris and Its Therapeutic Relevance in Acne Management. J. Dermatol. Treat..

[7] Hoover E., Aslam S., Krishnamurthy K. (2025). Physiology, Sebaceous Glands. StatPearls.

[8] Plewig G., Kligman A.M. (1978). Proliferative Activity of the Sebaceous Glands of the Aged. J. Investig. Dermatol..

[9] Zouboulis C.C. (2020). Endocrinology and Immunology of Acne: Two Sides of the Same Coin. Exp. Dermatol..

[10] Hong I., Lee M.-H., Na T.-Y., Zouboulis C.C., Lee M.-O. (2008). LXRα Enhances Lipid Synthesis in SZ95 Sebocytes. J. Investig. Dermatol..

[11] Zouboulis C.C., Jourdan E., Picardo M. (2014). Acne Is an Inflammatory Disease and Alterations of Sebum Composition Initiate Acne Lesions. J. Eur. Acad. Dermatol. Venereol..

[12] Picardo M., Ottaviani M., Camera E., Mastrofrancesco A. (2009). Sebaceous Gland Lipids. Dermatoendocrinology.

[13] Cunliffe W.J., Holland D.B., Jeremy A. (2004). Comedone Formation: Etiology, Clinical Presentation, and Treatment. Clin. Dermatol..

[14] Saint-Leger D., Bague A., Cohen E., Chivot M. (1986). A Possible Role for Squalene in the Pathogenesis of Acne. I. In Vitro Study of Squalene Oxidation. Br. J. Dermatol..

[15] Condrò G., Sciortino R., Perugini P. (2023). Squalene Peroxidation and Biophysical Parameters in Acne-Prone Skin: A Pilot In Vivo Study. Pharmaceuticals.

[16] O’Neill A.M., Gallo R.L. (2018). Host-Microbiome Interactions and Recent Progress into Understanding the Biology of Acne Vulgaris. Microbiome.

[17] Dreno B., Dekio I., Baldwin H., Demessant A.L., Dagnelie M.-A., Khammari A., Corvec S. (2024). Acne Microbiome: From Phyla to Phylotypes. J. Eur. Acad. Dermatol. Venereol..

[18] Sobhan M., Seif Rabiei M.A., Amerifar M. (2020). Correlation Between Lipid Profile and Acne Vulgaris. Clin. Cosmet. Investig. Dermatol..

[19] Jiang H., Li C.Y., Zhou L., Lu B., Lin Y., Huang X., Wei B., Wang Q., Wang L., Lu J. (2015). Acne Patients Frequently Associated with Abnormal Plasma Lipid Profile. J. Dermatol..

[20] Gökdemir G.Ş., Alp S., Nas C., Ecevit H. (2024). Evaluation of the Relationship between Lipid Profiles and Inflammatory Parameters in Patients with Acne Vulgaris. J. Med. Dent. Investig..

[21] Tribedi S., Tribedi S. (2024). Correlation between Acne Vulgaris Severity and Lipid Profile of the Patients Attending in a Tertiary Centre. Planet.

[22] Citra Utami O., Kurniawati Y., Diba S., Irsan Saleh M. (2019). Correlation between Serum Lipid Profile and Acne Vulgaris Severity. J. Phys. Conf. Ser..

[23] Akaza N., Akamatsu H., Numata S., Matsusue M., Mashima Y., Miyawaki M., Yamada S., Yagami A., Nakata S., Matsunaga K. (2014). Fatty Acid Compositions of Triglycerides and Free Fatty Acids in Sebum Depend on Amount of Triglycerides, and Do Not Differ in Presence or Absence of Acne Vulgaris. J. Dermatol..

[24] Smith R.N., Braue A., Varigos G.A., Mann N.J. (2008). The Effect of a Low Glycemic Load Diet on Acne Vulgaris and the Fatty Acid Composition of Skin Surface Triglycerides. J. Dermatol. Sci..

[25] Balić A., Vlašić D., Žužul K., Marinović B., Bukvić Mokos Z. (2020). Omega-3 Versus Omega-6 Polyunsaturated Fatty Acids in the Prevention and Treatment of Inflammatory Skin Diseases. Int. J. Mol. Sci..

[26] Aslan İ., Özcan F., Karaarslan T., Kıraç E., Aslan M. (2017). Decreased Eicosapentaenoic Acid Levels in Acne Vulgaris Reveals the Presence of a Proinflammatory State. Prostaglandins Other Lipid Mediat..

[27] Biełach-Bazyluk A., Jakubowicz-Zalewska O., Myśliwiec H., Flisiak I. (2026). Specialized Pro-Resolving Lipid Mediators and Dietary Omega-3/6 Fatty Acids in Selected Inflammatory Skin Diseases: A Systematic Review. Antioxidants.

[28] Guertler A., Neu K., Lill D., Clanner-Engelshofen B., French L.E., Reinholz M. (2024). Exploring the Potential of Omega-3 Fatty Acids in Acne Patients: A Prospective Intervention Study. J. Cosmet. Dermatol..

[29] Zhang L., Li Y., Pu Y., Dang T., Shi Q., Wu W. (2025). Exploring Clinical and Genetic Evidence in Association between Unsaturated Fatty Acids and Acne. Eur. J. Nutr..

[30] Liu M., Diaz-Torres S., Mitchell B.L., Toledo-Flores D., Gharhakhani P., Simpson M.A., Zhang H., Ong J.-S., Li J., Rentería M.E. (2025). The Role of Lipid Metabolism in Acne Risk: Integrating Blood Metabolite and Genetic Insights. Skin Health Dis..

[31] Melnik B. (2012). Dietary Intervention in Acne. Dermatoendocrinology.

[32] Khayef G., Young J., Burns-Whitmore B., Spalding T. (2012). Effects of Fish Oil Supplementation on Inflammatory Acne. Lipids Health Dis..

[33] Jeremy A.H.T., Holland D.B., Roberts S.G., Thomson K.F., Cunliffe W.J. (2003). Inflammatory Events Are Involved in Acne Lesion Initiation. J. Investig. Dermatol..

[34] Huang Y., Liu F., Lai J., Jiang S., Tan X., Chen L., Xu Y., Xiong X., Deng Y. (2024). The Adjuvant Treatment Role of ω-3 Fatty Acids by Regulating Gut Microbiota Positively in the Acne Vulgaris. J. Dermatol. Treat..

[35] Jung J.Y., Kwon H.H., Hong J.S., Yoon J.Y., Park M.S., Jang M.Y., Suh D.H. (2014). Effect of Dietary Supplementation with Omega-3 Fatty Acid and Gamma-Linolenic Acid on Acne Vulgaris: A Randomised, Double-Blind, Controlled Trial. Acta Derm. Venereol..

[36] Desbois A.P., Lawlor K.C. (2013). Antibacterial Activity of Long-Chain Polyunsaturated Fatty Acids against Propionibacterium Acnes and *Staphylococcus aureus*. Mar. Drugs.

[37] Claudel J.-P., Ballanger F., Auffret N., Leccia M.-T., Dréno B. (2025). Obesity: A Modulator in Acne Management. Acta Derm.-Venereol..

[38] Melnik B.C. (2018). Acne Vulgaris: The Metabolic Syndrome of the Pilosebaceous Follicle. Clin. Dermatol..

[39] Melnik B.C. (2015). Linking Diet to Acne Metabolomics, Inflammation, and Comedogenesis: An Update. Clin. Cosmet. Investig. Dermatol..

[40] Çerman A.A., Aktaş E., Altunay İ.K., Arıcı J.E., Tulunay A., Ozturk F.Y. (2016). Dietary Glycemic Factors, Insulin Resistance, and Adiponectin Levels in Acne Vulgaris. J. Am. Acad. Dermatol..

[41] Bungau A.F., Radu A.F., Bungau S.G., Vesa C.M., Tit D.M., Endres L.M. (2023). Oxidative Stress and Metabolic Syndrome in Acne Vulgaris: Pathogenetic Connections and Potential Role of Dietary Supplements and Phytochemicals. Biomed. Pharmacother..

[42] Juhl C.R., Bergholdt H.K.M., Miller I.M., Jemec G.B.E., Kanters J.K., Ellervik C. (2018). Dairy Intake and Acne Vulgaris: A Systematic Review and Meta-Analysis of 78,529 Children, Adolescents, and Young Adults. Nutrients.

[43] Dai R., Hua W., Chen W., Xiong L., Li L. (2018). The Effect of Milk Consumption on Acne: A Meta-Analysis of Observational Studies. J. Eur. Acad. Dermatol. Venereol..

[44] Sibi Krishna T., Kaur R., Malhotra V., Fatima Z., Mustajab M., Singh T., Iqbal M., Kesavan T., Ajeya S.P., Mathew S.G. (2025). The Impact of Isotretinoin on Lipid Profile: A Systematic Review. Ann. Med. Surg..

[45] Layton A. (2009). The Use of Isotretinoin in Acne. Dermatoendocrinology.

[46] Bagatin E., Costa C.S. (2020). The Use of Isotretinoin for Acne—An Update on Optimal Dosing, Surveillance, and Adverse Effects. Expert Rev. Clin. Pharmacol..

[47] Saklamaz A., Uyar B., Yalcin M., Cengiz H. (2016). Isotretinoin Increased Carotid Intima-Media Thickness in Acne Patients. Hippokratia.

[48] Kelhälä H.-L., Palatsi R., Fyhrquist N., Lehtimäki S., Väyrynen J.P., Kallioinen M., Kubin M.E., Greco D., Tasanen K., Alenius H. (2014). IL-17/Th17 Pathway Is Activated in Acne Lesions. PLoS ONE.

[49] Muscogiuri G., Cantone E., Cassarano S., Tuccinardi D., Barrea L., Savastano S., Colao A., on behalf of the Obesity Programs of Nutrition, Education, Research and Assessment (OPERA) Group (2019). Gut Microbiota: A New Path to Treat Obesity. Int. J. Obes. Suppl..

[50] Kershaw E.E., Flier J.S. (2004). Adipose Tissue as an Endocrine Organ. J. Clin. Endocrinol. Metab..

[51] González-Saldivar G., Rodríguez-Gutiérrez R., Ocampo-Candiani J., González-González J.G., Gómez-Flores M. (2017). Skin Manifestations of Insulin Resistance: From a Biochemical Stance to a Clinical Diagnosis and Management. Dermatol. Ther..

[52] Okoro O.E., Camera E., Flori E., Ottaviani M. (2023). Insulin and the Sebaceous Gland Function. Front. Physiol..

[53] Ozuguz P., Kacar S.D., Asik G., Ozuguz U., Karatas S. (2017). Evaluation of Leptin, Adiponectin, and Ghrelin Levels in Patients with Acne Vulgaris. Hum. Exp. Toxicol..

[54] Kaymak Y., Adisen E., Ilter N., Bideci A., Gurler D., Celik B. (2007). Dietary Glycemic Index and Glucose, Insulin, Insulin-like Growth Factor-I, Insulin-like Growth Factor Binding Protein 3, and Leptin Levels in Patients with Acne. J. Am. Acad. Dermatol..

[55] Ouchi N., Walsh K. (2007). Adiponectin as an Anti-Inflammatory Factor. Clin. Chim. Acta.

[56] Pischon T., Girman C.J., Rifai N., Hotamisligil G.S., Rimm E.B. (2005). Association between Dietary Factors and Plasma Adiponectin Concentrations in Men. Am. J. Clin. Nutr..

[57] Fantuzzi G. (2013). Adiponectin in Inflammatory and Immune-Mediated Diseases. Cytokine.

[58] Danby F.W. (2013). Turning Acne on/off via mTORC1. Exp. Dermatol..

[59] Ryguła I., Pikiewicz W., Kaminiów K. (2024). Impact of Diet and Nutrition in Patients with Acne Vulgaris. Nutrients.

[60] Melnik B.C., John S.M., Plewig G. (2013). Acne: Risk Indicator for Increased Body Mass Index and Insulin Resistance. Acta Derm.-Venereol..

[61] Leelambika C., Sarkar P. (2025). Evaluation of Lipid Profile Levels in Acne Vulgaris on Low Dose Isotretinoin: A Prospective Study. Int. J. Clin. Biochem. Res..

[62] Park Y.J., Shin H.Y., Choi W.K., Lee A.-Y., Lee S.H., Hong J.S. (2023). Optimal Laboratory Testing Protocol for Patients with Acne Taking Oral Isotretinoin. World J. Clin. Cases.

[63] Alajaji A., Alrawaf F.A., Alosayli S.I., Alqifari H.N., Alhabdan B.M., Alnasser M.A. (2021). Laboratory Abnormalities in Acne Patients Treated with Oral Isotretinoin: A Retrospective Epidemiological Study. Cureus.

[64] Nelson A.M., Gilliland K.L., Cong Z., Thiboutot D.M. (2006). 13-Cis Retinoic Acid Induces Apoptosis and Cell Cycle Arrest in Human SEB-1 Sebocytes. J. Investig. Dermatol..

[65] Khabour O.F., Alzoubi K.H., Firoz A.S., Al-Awad R.M. (2018). Association between Leptin Gene Rs7799039 Polymorphism and Lipid Profile Changes Induced by Isotretinoin Treatment in Acne Patients. Ther. Clin. Risk Manag..

[66] Wu L., Zhu S.-C., He Y., Zhu Y.-X., Ou-Yang X.-L., Zhang D., Li C.-M. (2025). Current Perspectives for Metabolomics and Lipidomics in Dyslipidemia of Acne Vulgaris: A Mini Review. Front. Med..

[67] Yu S., Xiao Z., Ou Yang X., Wang X., Zhang D., Li C. (2022). Untargeted Metabolomics Analysis of the Plasma Metabolic Signature of Moderate-to-Severe Acne. Clin. Chim. Acta.

[68] Su Q., Hu X., Yang M., He H., Jia Y. (2024). Lipidomic Analysis of Facial Skin Surface Lipids in Acne in Young Women. Int. J. Cosmet. Sci..

[69] Zhou M., Gan Y., He C., Chen Z., Jia Y. (2018). Lipidomics Reveals Skin Surface Lipid Abnormity in Acne in Young Men. Br. J. Dermatol..

[70] Zhou M., Yang M., Zheng Y., Dong K., Song L., He C., Liu W., Wang Y., Jia Y. (2020). Skin Surface Lipidomics Revealed the Correlation between Lipidomic Profile and Grade in Adolescent Acne. J. Cosmet. Dermatol..

